# Generalized arterial calcification of infancy and pseudoxanthoma elasticum: two sides of the same coin

**DOI:** 10.3389/fgene.2012.00302

**Published:** 2012-12-24

**Authors:** Yvonne Nitschke, Frank Rutsch

**Affiliations:** Department of General Pediatrics, Münster University Children’s HospitalMünster, Germany

**Keywords:** *ENPP1*, *ABCC6*, GACI, PXE, arterial calcification

## Abstract

Generalized arterial calcification of infancy (GACI) is associated with biallelic mutations in *ENPP1* in the majority of cases, whereas mutations in *ABCC6* (ATP-binding cassette subfamily C number 6) are known to cause pseudoxanthoma elasticum (PXE). However, *ABCC6* mutations account for a significant subset of GACI cases, and *ENPP1* mutations can also be associated with PXE lesions. Based on the considerable overlap of GACI and PXE, both entities appear to reflect two ends of a clinical spectrum of ectopic calcification rather than two distinct disorders. *ABCC6* and *ENPP1* mutations might lead to alterations of the same physiological pathways.

## GACI AND *ENPP1* MUTATIONS

Generalized arterial calcification of infancy (GACI; OMIM208000) is a rare autosomal recessive disease, which is characterized by severe calcification of the internal elastic lamina in large- and medium-sized arteries associated with intimal proliferation leading to arterial stenoses and heart failure within the first months of life. Although survival to adulthood has been reported, GACI is often lethal in the first 6 months of life. In the past, few patients survived the neonatal period (Moran, 1975; Morton, 1978), whereas more recently, patients treated with bisphosphonates have experienced a more favorable outcome (Rutsch et al., 2008; Ramjan et al., 2009). Some patients may also develop hypophosphatemic rickets with hyperphosphaturia, a finding associated with improved survival beyond infancy in patients with GACI (Rutsch et al., 2008; Levy-Litan et al., 2010; Lorenz-Depiereux et al., 2010). The disease has been found to be caused by inactivating mutations in *ENPP1* (MIM 173335; Rutsch et al., 2003). Mutations in *ENPP1* have been identified as the underlying defect in about 75% of the cases of GACI (Rutsch et al., 2008, 2011). *ENPP1* encodes the ecto-nucleotide pyrophosphatase/phosphodiesterase 1 (NPP1), a cell surface protein that catalyzes the hydrolysis of ATP to AMP and extracellular inorganic pyrophosphate (PP_i_; Rutsch et al., 2001; Goding et al., 2003). PP_i_ potently inhibits hydroxyapatite crystal deposition and growth and regulates chondrogenesis, collagen I expression and synthesis, and other cell differentiation processes (Bollen et al., 2000; Goding et al., 2003).

## PXE AND *ABCC6* MUTATIONS

Pseudoxanthoma elasticum (PXE; OMIM 264800) is a hereditary, autosomal recessive, multisystemic disease characterized by ectopic mineralization and fragmentation of elastic fibers of soft connective tissues such as the skin, the retina, and the arterial blood vessels. The clinical manifestations of classic PXE center on the skin, the eyes, and the cardiovascular system. The primary cutaneous lesions are small, yellowish papules on the neck and in large flexural areas, and these lesions progressively coalesce to form larger plaques, and skin folding occasionally develops. The eyes are frequently involved by calcification of Bruch’s membrane leading to angioid streaks, and bleeding from the choroidal vessels can result in loss of visual acuity and, occasionally, in central blindness. The cardiovascular manifestations derive from mineralization of arterial blood vessels, and include gastrointestinal bleeding, intermittent claudication, hypertension, and sometimes early myocardial infarcts. Additionally, PXE can manifest with gastrointestinal hemorrhage and abnormal tissue mineralization in different organs, including the liver, kidneys, spleen, breast, and testes (Li et al., 2009; Plomp et al., 2010). Although dermatological signs are common, the main burden of PXE results from the complications in the visual and cardiovascular systems (Hu et al., 2003). Cutaneous and eye involvement usually occurs in adolescence, but may appear earlier in childhood. Cardiovascular complications usually develop later, in mid-adulthood (Naouri et al., 2009). The prevalence of PXE is estimated to 1/25,000 to 1/75,000 in the general population (Chassaing et al., 2005; Li et al., 2009). Mutations in the *ABCC6* (ATP-binding cassette subfamily C number 6) gene are demonstrated in about 66–97% of patients who are genotyped (Bergen et al., 2000; Le Saux et al., 2000, 2001; Miksch et al., 2005; Chassaing et al., 2007; Vanakker et al., 2008). The ABCC6-transported substrate or substrates, which modulate arterial calcification and other phenotypic changes of PXE, are not known, and hepatic abnormalities that have effects on calcification-regulating plasma proteins such as fetuin have been suggested to at least partially mediate the pathogenesis of PXE (Hendig et al., 2006).

Generalized arterial calcification of infancy and PXE have been considered to be two distinct entities in the past and have been primarily linked to mutations in *ENPP1* and *ABCC6*, respectively. But recent findings indicate that GACI and PXE might be more closely related than previously thought.

## THE FIRST CASE OF GACI AND PXE IN ONE FAMILY

Recently, we reported on a family with two brothers born to unrelated parents. The elder developed uncomplicated PXE in adolescence. Interestingly, the younger brother died after his second myocardial infarction at 15 months of age. Autopsy demonstrated calcifications of the endocardium, with extensive calcifications of the coronary arteries and of medium-sized arteries and the aorta, leading to the diagnosis of GACI. We performed molecular genetic analyses in the family. Unfortunately, no DNA of the deceased younger brother with GACI was available. The elder brother had two heterozygous missense mutations of *ABCC6*. Each mutation was inherited from one of his heterozygous asymptomatic parents. However, no *ENPP1* mutations were found in the three living family members (Le Boulanger et al., 2010).

This case was the first one suggesting a correlation between PXE and GACI. We hypothesized that GACI could be independent of *ENPP1*, but related to *ABCC6* mutations and that on the other hand PXE could be related to *ENPP1* mutations.

## PATIENTS WITH GACI CARRY MUTATIONS IN *ABCC6*

Based on this case of GACI and PXE in one family with *ABCC6* mutations, we sequenced the *ABCC6* gene in 30 patients with a typical GACI phenotype but without disease-causing *ENPP1* mutations. In 14 of these patients, we detected pathogenic mutations in *ABCC6* (biallelic mutations in eight patients, monoallelic mutations in six patients). This study showed that biallelic mutations in the *ABCC6* gene account for a substantial number of typical GACI cases (Nitschke et al., 2012). The fact that even monoallelic mutations in *ABCC6* were associated with the severe phenotype of GACI cannot fully be explained on the basis of autosomal recessive inheritance. However, mutations of other disease-associated genes have not been ruled out so far.

## PATIENTS WITH GACI AND PXE CARRY MUTATIONS IN *ENPP1*

Three of our GACI patients, who showed extensive calcifications of the large- and medium-sized arteries, arterial stenoses, and periarticular calcifications in infancy, carried biallelic *ENPP1* mutations. These patients developed clinical features of PXE in childhood between 5 and 8 years of age. The patients showed angioid streaks and typical pseudoxanthomatous skin lesions (Nitschke et al., 2012). Most recently, one additional 2-year-old patient with a relatively mild form of GACI developed PXE with pseudoxanthomatous lesions of the neck, inguinal folds, and lower abdomen. The patient was also found to harbor a homozygous missense mutation in *ENPP1* (Li et al., 2012).

## GENOCOPY AND PHENOCOPY IN GACI AND PXE

**FIGURE 1:**
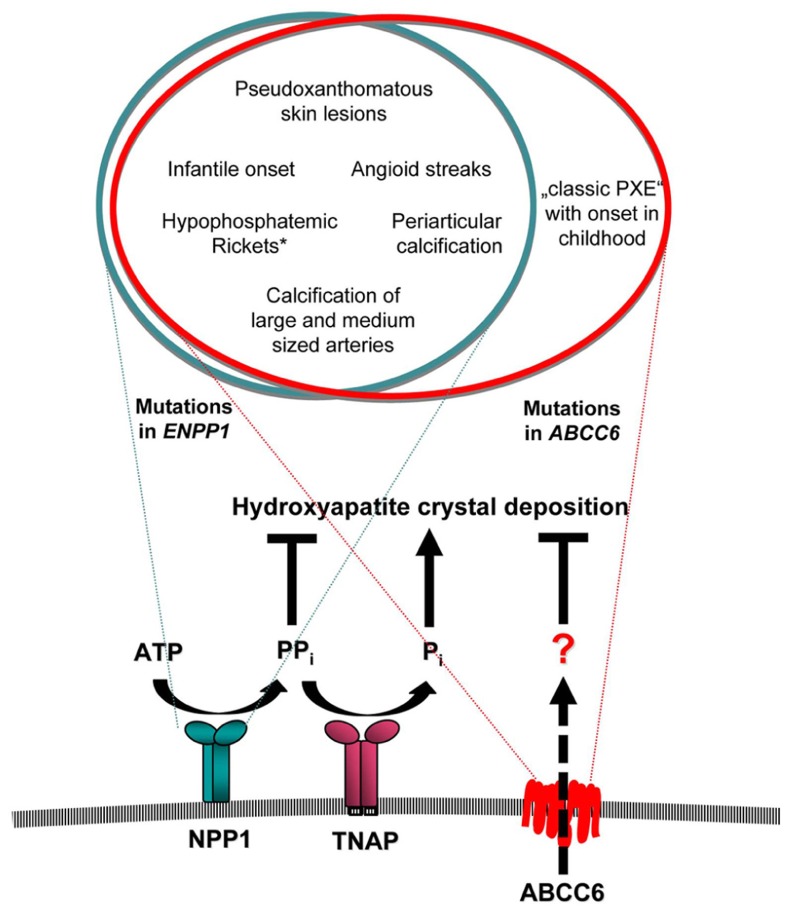
**Overlap of clinical manifestations associated with mutations in *ENPP1* and *ABCC6*.** NPP1 and ABCC6 serve as a minor component in the function of a network of factors that exert balanced effects to promote and suppress arterial calcification. The transmembrane ectoenzyme NPP1 generates AMP and PP_i_ from ATP. PP_i_ is hydrolyzed by the tissue non-specific alkaline phosphatase (TNAP) to generate P_i_, which is a component of hydroxyapatite crystal deposition and plays a role in the regulation of osteoblast differentiation. PP_i_ suppresses hydroxyapatite deposition and inhibits ectopic chondrogenesis (and modulates artery calcification by other effects). The role of ABCC6 has to be defined. Mutations of either *ABCC6* or *ENPP1* can cause the severe phenotype of GACI, which frequently leads to death within the first year of life. While mutations in *ENPP1* can also cause typical pseudoxanthomatous skin lesions and angioid streaks of the retina in children with GACI, who survived the critical period of infancy, the later onset of “classic PXE” phenotype without GACI was only observed in patients with mutations in *ABCC6*. *Hypophosphatemic rickets has been observed frequently in patients with *ENPP1* mutations, but was observed only in one proband carrying a mutation in *ABCC6* on one allele.

GACI and PXE have been considered to be two distinct entities in the past and have been primarily linked to *ENPP1* and *ABCC6*, respectively. But based on the overlap of genotype and phenotype of GACI and PXE, both entities appear to reflect two ends of a clinical spectrum of ectopic calcification and other organ pathologies, rather than two distinct disorders (**Figure 1**). It was shown, that biallelic mutations in *ABCC6* account for a significant number of typical GACI cases, which involve widespread arterial calcifications, arterial stenoses, periarticular calcifications, and hypophosphatemic rickets. *ABCC6* mutations can be associated with a much more severe phenotype, including death in infancy from myocardial infarction, than was previously known. We conclude that the phenotypic spectrum of diseases associated with *ABCC6* mutations is much broader than was previously assumed. In fact, the infantile phenotype of patients carrying *ABCC6* mutations can be indistinguishable from the phenotype associated with *ENPP1* mutations. The fact that the same *ABCC6* mutations can cause the severe GACI phenotype associated with death in early infancy and the relatively mild phenotype of PXE warrants further explanation. Because of the difficulty with charting a clear pattern of inheritance to phenotype, it is likely that mutations in other disease-associated genes may play a role here.

Up to date, four patients who presented with GACI and carried biallelic *ENPP1* mutations developed the clinical manifestation of PXE in childhood. Symptoms included angioid streaks and histologically proven calcifications of elastic skin fibers. Thus, given the poor prognosis of severe GACI, affected patients might die of the cardiovascular complications of the disease before they develop typical signs of PXE. This might be the reason that no previous case of GACI has been described in the PXE literature. Also, many PXE characteristics, including angioid streaks of the retina and peau d’orange skin lesions might frequently be overlooked in the clinical examinations of GACI patients. Hence, the true number of patients carrying *ENPP1* mutations and showing PXE lesions might be higher. In summary, these findings show that mutations in the different genes *ENPP1* and *ABCC6* can lead to similar pathophysiological consequences and that GACI and PXE do not simply represent two distinct disorders. They rather represent a spectrum of different peculiarities of ectopic calcification. It can therefore be hypothesized that the pathophysiology of *ENPP1* and *ABCC6* related disorders is based on common downstream mechanisms.

## Conflict of Interest Statement

The authors declare that the research was conducted in the absence of any commercial or financial relationships that could be construed as a potential conflict of interest.
